# Highly stable mesoporous Co/Ni mixed metal-organic framework [Co/Ni(μ3-tp)_2_(μ2-pyz)_2_] for Co (II) heavy metal ions (HMIs) remediation

**DOI:** 10.1016/j.heliyon.2024.e35044

**Published:** 2024-07-23

**Authors:** Ehsan Moradi, Mohammad Mehdi Salehi, Ali Maleki

**Affiliations:** Catalysts and Organic Synthesis Research Laboratory, Department of Chemistry, Iran University of Science and Technology, Tehran, 16846-13114, Iran

**Keywords:** MOF, Co (II), Mesoporous, Heavy metal ions, Elimination, Adsorption

## Abstract

A bimetallic cobalt/nickel-based metal-organic framework (MOF), [Co/Ni(μ3-tp)_2_(μ2-pyz)_2_], denoted as Co/Ni-MOF, has been successfully prepared by a hydrothermal method. The MOF was prepared by incorporating mixed O^−^ and N^−^ donor ligands, specifically terephthalic acid (tp) and pyrazine (pyz). The Mesoporous Co/Ni-MOF was comprehensively characterized using various analytical methods such as XRD, BET, FT-IR, TGA (23 % char yields), SEM, and EDS analyses. The synthesized mesoporous Co/Ni-MOF was then used to absorb Co (II) from aquatic areas efficiently. Several critical parameters, such as the beginning Co (II) concentration (25–150 mg/L), the effect of pH (2–10), the duration of time (5–30 min), and the amount of adsorbent (0.003–0.02 g), were systematically investigated. Remarkably, the Mesoporous Co/Ni MOF displayed a significant adsorption capacity of 372.66 mg g^−1^ in the optimum conditions, including pH = 6, amount of adsorbent = 0.003 g, duration of time = 25 min, and beginning Co (II) concentration = 150 mg/L. Adsorption data from the experimental studies of the mesoporous Co/Ni MOF are matched based on the non-linear pseudo-first-order (PSO) kinetic model (R^2^ = 0.9999), and a chemical process is suggested for chemisorption. Furthermore, the adsorption isotherms of Co (II) heavy metal ions (HMIs) are an excellent fit with the non-linear Temkin, indicating that they explain the sorbent/sorbate interactions concerning the heat of adsorption. It is evident from the thermodynamic parameters that adsorption is a spontaneous and favorable exothermic process. These results highlight the promising adsorption performance and potential applications of the mesoporous Co/Ni-MOF as an effective adsorbent for Co (II) elimination from aquatic areas. Four-cycle regeneration studies were the most effective for the Co (II) under study.

## Highlights

• Co/Ni-based MOF prepared successfully by hydrothermal methods.
• Adsorption data were fitted by Freundlich isotherms and pseudo second-order kinetics.
• The Mesoporous Co/Ni MOF preparation reused at least four more times.

## Graphical abstract

**Figure:**
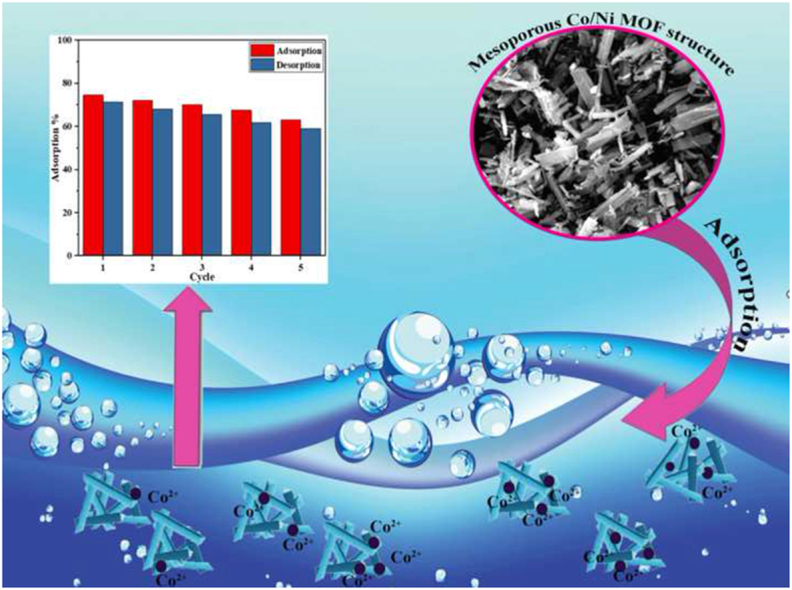

## 1 Introduction

**Fig. 1:**
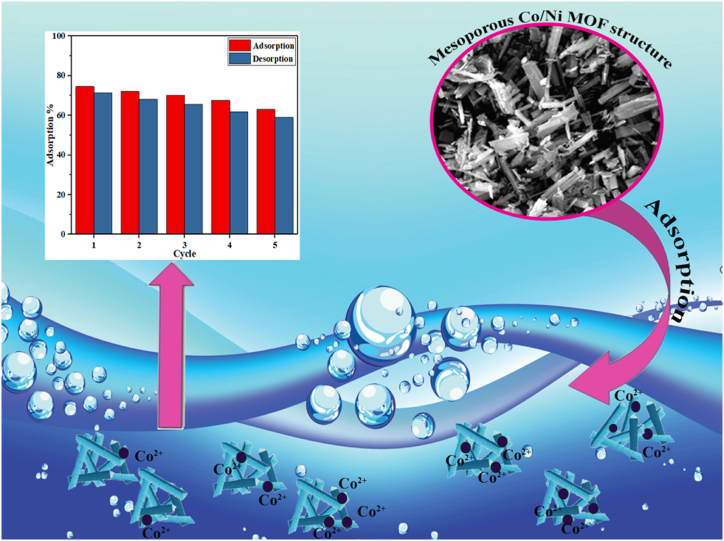
Graphical abstract of heavy metal remediation by mesoporous Co/Ni Mixed Metal-Organic Framework.

Growing concerns about pollution and global freshwater scarcity threaten human health and ecosystems [[1], [2], [3], [4]]. Of particular concern is the contamination of water and wastewater with pesticides [5], toxic dyes [6,7], antibiotics [8], and heavy metal ions (HMIs) [[9], [10], [11]], which has emerged as an urgent ecological crisis [[12], [13], [14]]. Industries operating in countries with inadequate or non-existent regulatory frameworks often discharge metalliferous effluents into freshwater without proper treatment. This irresponsible discharge of metalliferous wastewater contributes to the accumulation of heavy metals in water resources. In addition, human activities, such as burning fossil fuels, industrial processes, agricultural practices, and dietary habits, exacerbate this problem, resulting in adverse environmental impacts. HMI pollution adversely affects the anthroposphere's water, air, and soil components. HMIs, including lead, mercury, cadmium, arsenic, chromium, cobalt, selenium, nickel, copper, and zinc, are highly toxic, tend to bioaccumulate, pose severe risks to human health and natural ecosystems, and cause significant environmental problems [15,16]. HMIs are released into the environment through various natural processes, such as spring water, erosion, volcanic eruptions, and bacterial activity. While these HMIs are typically present in trace amounts in natural water sources, many are toxic at deficient concentrations. In addition to osteoporosis, renal damage, bronchiolitis, cardiovascular disease, and even cancer, several diseases have been proven to be associated with long-term exposure to HMIs [17,18]. Among these HMIs, cobalt (II) is particularly toxic, and its excessive intake has been linked to health problems, including asthma, allergies, heart failure, and damage to the liver and thyroid. To release Co (II) effluents into the environment, those effluents should be effectively purified. Various methods are employed to remove HMIs from aqueous solutions, including electrochemistry, membrane separation, ion exchange, chemical precipitation, and adsorption. The adsorption method is among the most prominent and effective techniques for removing HMIs from wastewater [17,19,20]. Researchers have reported using various adsorptive materials to treat HMIs wastewater in the last few decades, such as biomass adsorbents, inorganic minerals, activated carbon, metal-organic frameworks (MOFs), polymers, and others. Surface-active functional groups and structural features of adsorbents determine their ability to absorb HMIs [18,21,22]. Through chemical or physical interaction with adsorbents, HMIs attach to their surface-active sites. There are several mechanisms by which adsorbents remove HMIs from aqueous media, including physical adsorption, electrostatic interaction, coordination interaction, and ion exchange [23]. As a result of their unique properties, specifically high porosity, large specific surface area, and tunable structure, MOFs have attracted considerable attention in recent years [[24], [25], [26], [27], [28]]. These properties make MOFs highly versatile for various applications, including gas storage [[29], [30], [31]], gas separation [32,33], catalysis [34,35], drug delivery [36,37], sensing [[38], [39], [40]], and adsorption [26,[41], [42], [43], [44]]. The unique properties of MOFs, particularly their well-defined pores and channels, allow target compounds to access the active sites within the MOF crystals efficiently [45]. This makes MOFs a promising adsorption material with the highest performance quality in removing toxic and radioactive HMIs [[46], [47], [48], [49]]. Bimetallic MOFs are a remarkable type of porous material containing two metal sources and organic ligands [[50], [51], [52]]. In particular, Co/Ni bimetallic MOFs are characterized by excellent hydrophilicity and stability [[53], [54], [55]]. Due to the promising properties of MOFs, they have emerged as potential candidates for heavy metal removal applications. Motaghi et al. investigated the adsorption performance of Co (II) in water using the bio-nanocomposite MOF MCS/AC@UiO-66 and achieved a remarkable adsorption efficiency of 44.5 % within 15 min. Wang et al. proposed microporous zeolitic imidazole framework-90, obtained by post-synthetic modifications of ZIF-90-Met and ZIF-90-Lys, for Co (II) removal. The maximum adsorption capacities of Co (II) on ZIF-90-Met and ZIF-90-Lys were reported to be 136.83 and 164.40 mg g^−1^, respectively, for a period of 80 min. Yuan et al. investigated the adsorption of Co (II) from wastewater using MOFs. The synthesized MOF adsorbents, namely UiO-66-COOCH_3_, UiO-66-CONH_2_, UiO-66-CN, and UiO-66-SO_3_H, exhibited exceptional adsorption performance with saturated adsorption capacities of 334.4, 339.7, 274.6, and 293.7 mg g^−1^, respectively. Moreover, Zhuo et al. synthesized ZIF-90-SO_2_HN_2_, which showed an optimal adsorption capacity of about 122.85 mg g^−1^ for Co (II) below specific conditions (pH = 6.72, T = 303 K, C0 = 250 mg L^−1^) (see Fig. 1).

**Fig. 2:**
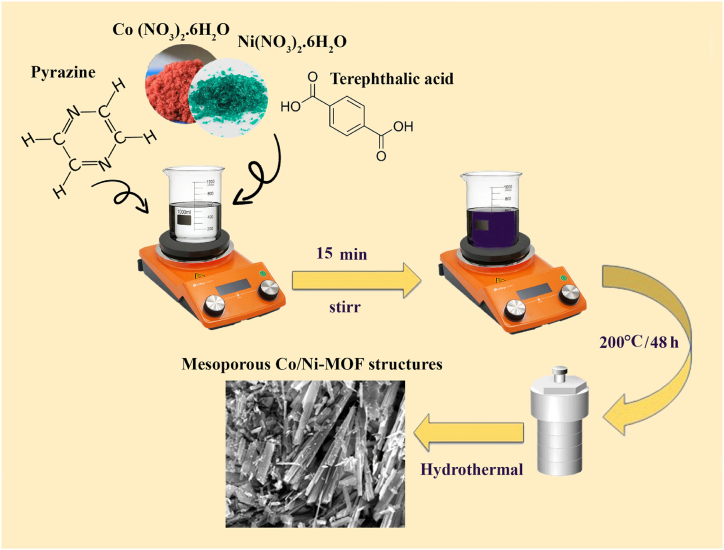
Schematic preparation of mesoporous Co/Ni MOF.

This study aims to improve the properties of adsorption materials to eliminate Co (II) ions from aquatic areas efficiently. We modified the synthesis to develop terephthalic acid-functionalized MOF materials based on a Co/Ni bimetallic framework via a hydrothermal reaction to achieve this. The resulting Co/Ni bimetallic-based MOFs, designated as [Co/Ni(μ3-tp)_2_(μ2-pyz)_2_] (Co/Ni-MOF) (tp = terephthalic acid and pyz = pyrazine), were successfully synthesized as illustrated in Fig. 2. Subsequently, the applicability of these Co/Ni bimetallic-based MOFs for eliminating HMIs from water was researched, with a particular focus on the elimination of Co (II) ions. The Co/Ni MOFs exhibited exceptional performance in eliminating Co (II) ions from aquatic areas, demonstrating their potential as effective adsorbents. We analyzed the adsorption isotherms and kinetics to gain insight into the process and investigated the underlying adsorption mechanism. In addition, we explored the beneficial application of Co/Ni MOFs in the adsorption of Co (II) ions in wastewater, highlighting their potential in addressing environmental contamination issues.

## 2 Experimental section

### 2.1 Materials and instruments

All of the materials and apparatus used in this work have been summarized in Table S1.

### 2.2 Synthesis of the mesoporous Co/Ni-MOF compound

Briefly, we have prepared a stable and high-surface-area metal-organic framework (MOF) based on cobalt and nickel metal nodes with terephthalic acid and pyrazine linkers with the help of previously reported work [54]. Co/Ni MOF was synthesized by the hydrothermal reaction method using a mixture containing Co (NO_3_)_2_.6H_2_O (0.18901 g or 1 mmol), Ni (NO_3_)_2_.6H_2_O (0.18877 g or 1 mmol), terephthalic acid (0.33228 g or 2 mmol), pyrazine (0.1602 g or 2 mmol), and 15 mL of distiller water (d.w.). The resulting suspension was stirred for 5 min and then transferred to a 25 mL stainless steel Teflon reactor autoclave. The autoclave was heated to 200 °C and maintained at this temperature for 48 h. The reaction mixture was then cooled gradually to room temperature (r.t), and a filter was applied to the resulting product. The filter was then washed with d.w. And dried in r.t.

### 2.3 The batch adsorption process: experimental studies

Co (II) adsorption by Co/Ni-MOF was carried out at r.t. The effect of various adsorption conditions, such as beginning ion concentration (25–150 ppm), duration time (5–30 min), amount of adsorbent (0.003–0.02 g), and effect of pH (2–10), was investigated. Aqueous solutions containing cobalt ions at different concentrations were prepared using cobalt (II) nitrate. A predetermined amount of Co/Ni-MOF was added to 50 mL of cobalt (II) solution to initiate the adsorption process. The resulting mixture was then stirred at 550 rpm for 20 min. The pH of each solution was adjusted in the range of 3–9 by using HCl and NaOH (both 0.1M). Adsorption isotherms were also determined by comparing linear and non-linear a variety of isotherms, including Freundlich, Langmuir, Temkin, and Redlich Peterson models with experimental data. The adsorption kinetics were also evaluated using linear and non-linear kinetics such as pseudo-first-order (PFO), pseudo-second-order (PSO), Weber-Morris Intraparticle Diffusion, and Elovich models. Over a period of time, the mesoporous Co/Ni MOF was separated from the solution by centrifugation. ICP-OES then measured the solution's final concentration of Co (II). This measurement allowed a quantitative analysis of the concentration of cobalt (II) ions remaining in the solution after adsorption. Three replicated experiments were conducted, and the average results were calculated to minimize errors [56]. Using Eqs. (1), (2), (3)), the capacity and efficiency of Co (II) adsorption were calculated on a mesoporous material made of Co/Ni MOF:

(1) $$\%\;\text{Adsorbtion}=\left(\frac{\text{Ci}-\text{Ce}}{\text{Ci}}\right)\times 100$$

(2) $$Qe=\left(\frac{Ci-Ce}{m}\right)\times V$$

(3) $$R\;\left(\%\right)=\left(C_{0}–C_{t}\right)/C_{0}\times 100$$

It is reported that in aqueous solutions, Co (II) is present at one equilibrium concentration (mg/L) and one initial concentration (mg/L). Assuming (m) represents the weight of the mesoporous Co/Ni MOF, (L) represents the volume of the solution.

### 2.4 An investigation of regeneration and reusability

It also provides information on recycling adsorbent materials, which helps reduce wastewater treatment costs. A four-cycle regeneration was conducted to evaluate mesoporous Co/Ni-MOF adsorbent reusability for Co (II) adsorption. The results are located in Fig. S1. Increasing regeneration cycles decreased adsorption efficiency (R%) from 74.53 % to 62.98 % when regeneration cycles were increased four times. Prior literature showed that increasing regeneration cycles reduced adsorption efficiency. It is thus possible to reuse mesoporous Co/Ni-MOF for adsorption of Co (II) HMIs up to four times based on this design. According to the results, mesoporous Co/Ni-MOF is an economically feasible and stable solution for removing Co (II), making it suitable for treating a variety of HMIs [57]. Eq (4) were used to calculate the desorption percentage (D%):

(4) $$D\left(\%\right)=\frac{A\;}{B}\times 100$$

(A) describes several contaminants as part of the renewal solution, desorbed (mg), and (B) represents several contaminants in the solution for rinsing (mg).

## 3 Results and discussion

The [Co/Ni(μ3-tp)_2_(μ2-pyz)_2_] MOF was prepared by hydrothermal methods by combining cobalt nitrate, nickel nitrate, terephthalic acid, pyrazine, and d.w as a solvent. The synthesis process was carried out for 48 h. The resulting mesoporous Co/Ni MOF compound has a 3D network structure with porous properties, which can be adjusted to form a 1D channel by double interpenetration. In the process of complexing, Nitrogen-rich Chelating Ligands (Pyrazine), nitrogen atoms are strongly chelated with Co (II) ions. The transition metal ions accept electrons from these free electron pairs.

### 3.1 Structural characterization of Co/Ni-MOF

#### 3.1.1 Philips X-pert diffractometer (PXRD) analysis

**Fig. 3:**
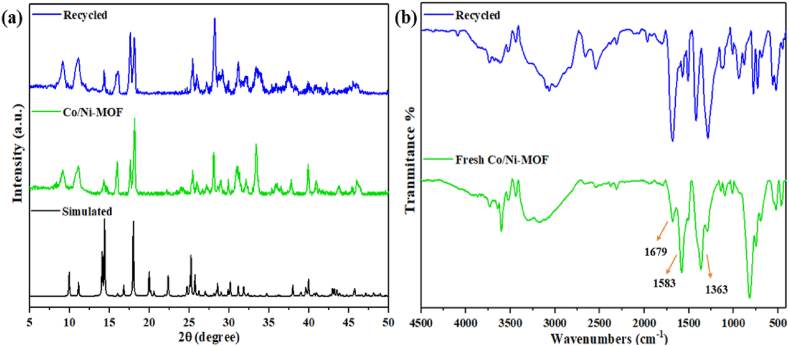
**(a)** PXRD pattern of as-synthesized, simulated, and recycled mesoporous Co/Ni-MOF, and **(b)** FT-IR spectra of mesoporous Co/Ni-MOF, and recycled spectra of Co/Ni-MOF.

In the asymmetric unit of mesoporous Co/Ni MOF, there is one H_2_bdc anion, one pyrazine molecule, half of a Co (II) cation, and half of a Ni (II) cation. H_2_bdc ligand is coordinated to two oxygen atoms by Co^2+^ and Ni^2+^ centers, exhibiting a bidentate coordination mode, and two oxygen atoms of two dissimilar terephthalic acid ligands, exhibiting a bridging bis-monodentate coordination mode. This coordination arrangement has been reported in the literature. The XRD pattern of the hydrothermally synthesized mesoporous Co/Ni MOF, the recycled material, and the simulated pattern are shown in Fig. 3a, according to references [53,54].

#### 3.1.2 Fourier-transform infrared spectroscopy (FT-IR) analysis

FTIR analysis was carried out to identify the samples in terms of their functional groups. In Fig. 3b, we have shown the FT-IR spectrum for the fresh mesoporous Co/Ni MOF and the recycled material. Asymmetric stretching vibrations are observed at the wavenumbers of 1583 and 1679 cm^−1^, whereas symmetric stretching vibrations of carboxylate groups are observed at the wavenumbers of 1363 and 1583 cm^−1^, respectively. As can be seen from the splitting of the asymmetric stretching band, two modes of coordination exist between the carboxylate groups. Complete deprotonation of the terephthalic acid ligands within Mesoporous Co/Ni-MOF is further confirmed by the lack of bands for protonated carboxylate groups at 1690-1730 cm^−1^. Mesoporous Co/Ni-MOF exhibits two different modes of coordination based on FT-IR analysis, which is consistent with the spectrum of other compounds that contain terephthalic acid ligands and exhibit two modes of coordination [58,59].

#### 3.1.3 Thermogravimetric analysis (TGA)

**Fig. 4:**
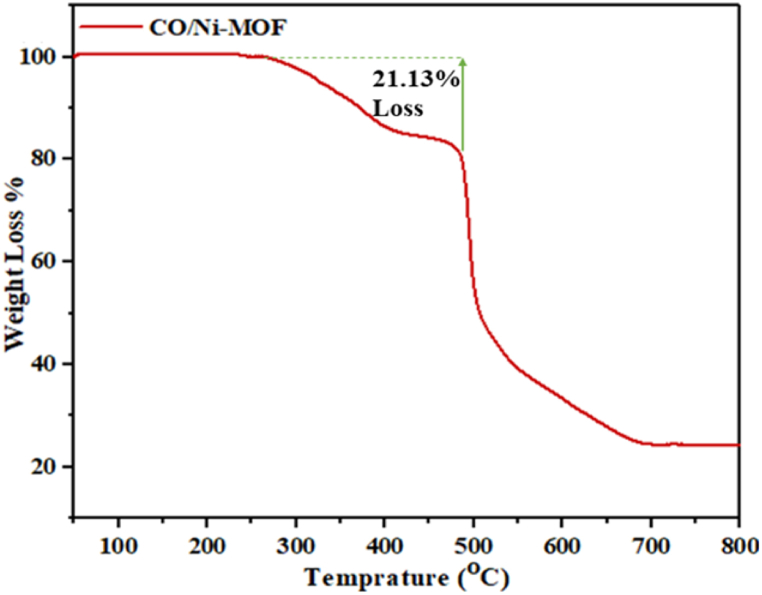
TGA of activated mesoporous Co/Ni-MOF.

TGA evaluated the thermal stability of the mesoporous Co/Ni MOFs. Fig. 4 shows that these samples lose weight under an N_2_ atmosphere according to temperature. As the temperature increases to 420 °C, the weight loss increases to 21.13 %. It is possible to attribute this weight loss to the evaporation of H_2_O molecules from the guest solvent (about 5 %) between 50 and 350 °C, a phenomenon observed in other MOFs synthesized using water as a solvent. The TGA curve obtained for Mesoporous Co/Ni MOF in this temperature range is similar to that reported for this MOF, indicating its high thermal stability. This thermal stability can be attributed to the coordinated interpenetrating framework of the MOF structure [60].

#### 3.1.4 Scanning electron microscopy (FESEM)

**Fig. 5:**
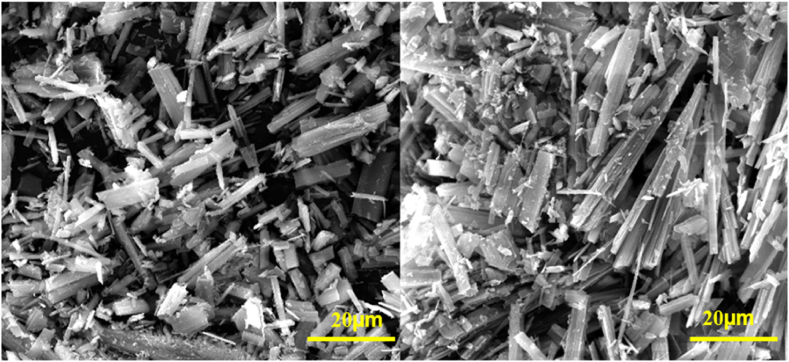
SEM images of mesoporous Co/Ni-MOF structures prepared by the hydrothermal method.

We identified the mode of particle aggregates, size distributions, and microstructure morphology of prepared samples using FESEM under the guidelines in Fig. 5. SEM examined the morphologies of the synthesized mesoporous Co/Ni MOFs. As shown in Fig. 5, the MOFs exhibit a symmetric geometry with a cuboid rod-like morphology.

#### 3.1.5 Brunauer-emmett-teller (BET)

**Fig. 6:**
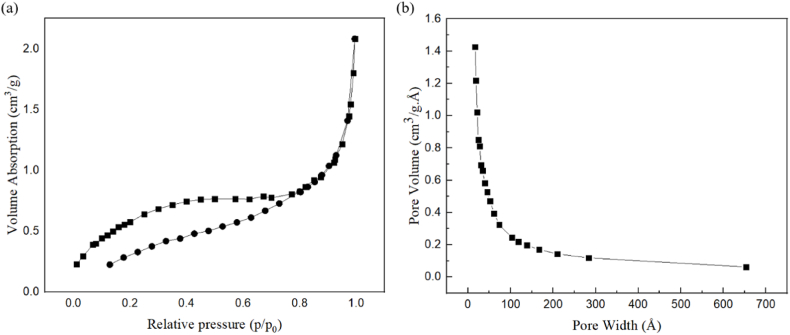
An adsorption-desorption isotherm is shown in panel **(a)** for mesoporous Co/Ni-MOFs made of N_2_. A mesoporous Co/Ni-MOF is shown in panel **(b)** with a distribution of pore sizes.

As illustrated in Fig. 6, the theory of nitrogen gas adsorption and desorption was investigated as a technique to measure specific surface area, pore volume, and pore size using mesoporous Co/Ni-MOF [61]. Based on Barrett, Joyner, and Halenda (BJH) theory, the BET surface area, pore volume, and average pore diameter of mesoporous Co/Ni-MOF are summarized in Table S1. In mesoporous Co/Ni-MOF, a hysteresis loop is rarely observed in adsorption/desorption isotherms of 0.45–1.0 p/p0, indicating that these materials have holes (Type IV). Table S1 shows the results of BET. The surface area, pore volume, and pore size of mesoporous Co/Ni-MOF were measured at 2.3070 m^2^ g^−1^, 0.0022 cm^3^ g^−1^, and 38.7632 nm, respectively.

### 3.2 Adsorption experiments

#### 3.2.1 Performance of mesoporous Co/Ni-MOF for Co (II) adsorption

Combining all these factors contributed to eliminating contamination from aquatic areas using adsorbents, including the effect of pH, duration of time, amount of adsorbent, and beginning concentration. By adjusting these parameters in a precise and efficient manner, it is possible to increase the efficiency of the contaminant removal process very effectively [62].

#### 3.2.2 Effect of pH

**Fig. 7:**
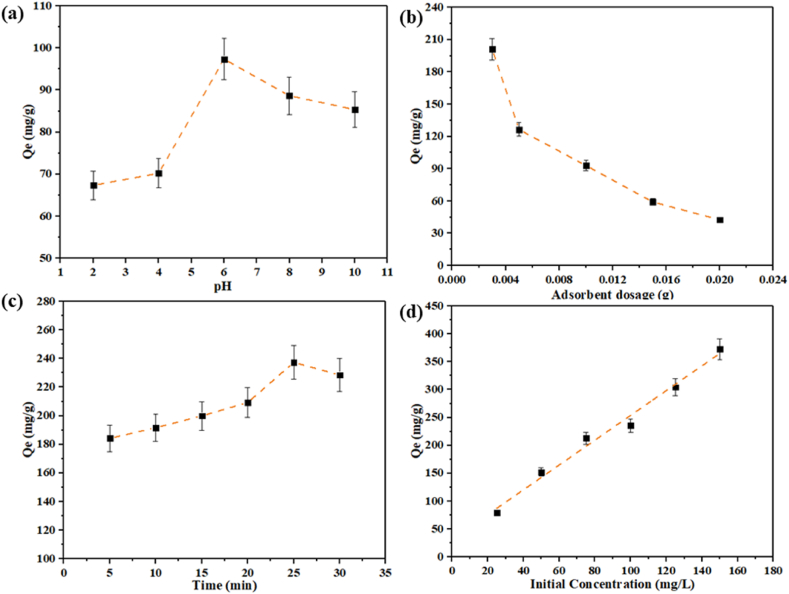
**(a)** Effect of pH; **(b)** amount of adsorbent; **(c)** duration of time; **(d)** beginning concentration.

The pH range (2–10) significantly affects the adsorption process. First, a 50-ppm Co (II) solution was prepared to study the adsorption performance of mesoporous Co/Ni-MOF. In separate vessels, 10 mg of the adsorbent was added to 50 mL of the cobalt-containing solution. Fig. 7a illustrates the effect of pH on the adsorption of Co (II) ions. Under acidic conditions, the removal efficiency of Co (II) ions decreases due to competition among metal ions and hydronium ions (H_3_O^+^) for binding to the mesoporous Co/Ni MOF. However, as the pH range increases and the concentration of H3O^+^ decreases, electrostatic solid interactions occur between Co (II) and the mesoporous Co/Ni MOF. Based on the various pH (2–10) results, the highest Co (II) adsorption rate by mesoporous Co/Ni MOF was observed at pH 6 with an adsorption capacity of 97.37 mg g^−1^. Therefore, subsequent experiments were performed at this pH.

#### 3.2.3 Amount of adsorbent

Various mesoporous Co/Ni MOF amounts were used to investigate the relationship between the mesoporous amount and Co (II) adsorption capacity. The mesoporous Co/Ni MOF for Co (II) adsorption capacity decreased from 201.23 mg g^−1^ to 42.67 mg g^−1^ when the adsorbent dosage was increased from 0.003 to 0.02 g, as illustrated in Fig. 7b. Due to the high contamination amount available for the mesoporous Co/Ni MOF, Co (II) can be adsorbed with a large amount at lower adsorbent dosages; as a result, lower dosages of adsorbent provide enhanced adsorption capabilities. The best adsorbent dosage for further evaluation was 0.003 g of mesoporous Co/Ni MOF.

#### 3.2.4 Duration of time

From 5 to 30 min at optimal pH and amount of adsorbent, the duration impact on mesoporous Co/Ni MOF adsorption of Co (II) was studied. Accordingly, by increasing the time to 25 min, Co (II) adsorption capacity increases by 237.43 mg g^−1^ (Fig. 7c). Although adsorption capacity does not decrease dramatically, it does decrease slightly to 30 min. Accordingly, Co (II) should be contacted for 25 min at an optimum pH and amount of adsorbent. During the adsorption reaction, the number of unoccupied active sites in the mesoporous Co/Ni MOF results in a rapid mass transfer due to the large number of unoccupied active sites. It takes approximately 25 min of the reaction process for the mesoporous functional groups to interact efficiently with Co (II). As a result, no adsorption capacity improvement was observed after reaching the equilibrium maximum adsorption capacity of mesoporous Co/Ni MOFs.

#### 3.2.5 Beginning concentration

A mesoporous Co/Ni MOF was found to have enhanced adsorption capabilities when the Co (II) concentration was increased to 150 mg L^−1^. In this case, pH (6), adsorbent amount (0.003 g), and duration of time (25 min) were optimized. As shown in Fig. 7d, the adsorption capacity of the mesoporous was significantly affected by the concentration of Co (II) at the beginning of the experiment and by increasing the concentration of Co (II) from 25 to 150 mg L^−1^, the adsorption capacity was enhanced. The amount of Co (II) adsorbate was enhanced by altering the starting concentrations while maintaining an equal amount of adsorbent, thus increasing mesoporous Co/Ni MOF adsorption capacity.

### 3.3 Study of isotherms

To characterize and optimize adsorption systems, it is necessary to understand how adsorbate (contaminants) and adsorbent interact. Various adsorption isotherm studies are conducted to determine surface properties, maximum uptake capacity, and mechanisms. To analyze the HMI uptake behavior and surface binding affinity of the mesoporous Co/Ni-MOF biosorbent, equilibrium adsorption experiments were performed under optimal pH, amount of adsorbent, duration of time, and concentration beginning conditions. Several popular and influential isotherm models were used to fit the experimental equilibrium data – Langmuir, Freundlich, Temkin, and Redlich-Peterson in both their linear and non-linear forms. Based on regression analysis, Table S5 details the equations defining the models and the parameters derived from the models.

Fig. S2 and Table S4 show that, among all models, the non-linear Temkin isotherm offered the best fit with the highest coefficient of determination (R^2^ = 1.00) as opposed to the Langmuir, Freundlich, and Redlich-Peterson models. As a result of the superior agreement of the Temkin isotherm, pesticide adsorption is primarily a chemisorption process. The Temkin isotherm determines the interactions between the adsorbate substances and the adsorbent and the bonding energies involved in the adsorption process. The adsorption energy between the adsorbent's covered surface and the adsorbate decreased based on the descending linear pattern. An adsorbent's surface coating function represents the free energy of the adsorption procedure in the Temkin model. The Temkin fitting also indicates that binding energies are distributed uniformly up to a maximum, which aligns with equilibrium experiments that observe gradual saturation [5,63,64].

### 3.4 Study of kinetics

Understanding the pathways, rate-controlling mechanisms, and time required to achieve interfacial equilibrium is crucial to understanding the kinetic behavior of Co (II) adsorption on the mesoporous Co/Ni-MOF biosorbent. Four important kinetic models fit these experimental data: pseudo-first-order (PFO), pseudo-second-order (PSO), Weber-Morris intraparticle diffusion, and Elovich models. As summarized in Table S5, all model equations and parameters were analyzed for both linear and non-linear models. Models are provided to provide interpretations of the chemical and physical interactions that contribute to adsorption, distinguishing between sorption at the surface of the particles and diffusion within the particles (Fig. S3). In comparing coefficients of determination (R^2^) values and agreement between experimental and calculated Qe values, the non-linear PFO model was the best fit with an R^2^ = 0.999. Using this kinetic model, we can determine the heterogeneous adsorbents involved in adsorption; however, it does not predict conventional mechanisms. Mesoporous Co/Ni-MOF biosorbent surfaces retain heterogeneous active sites due to HMIs predicted onto the surface during the total adsorption time, indicating adsorption kinetics [65].

### 3.5 Adsorption thermodynamics

To understand the mechanisms underlying Co (II) elimination from a mesoporous Co/Ni MOF adsorbent, thermodynamic studies offer comprehensive insights into the intrinsic energetic changes employed during Co (II) removal. Co (II) adsorption was studied over a mesoporous Co/Ni MOF adsorbent at various temperatures, and the Gibbs free energy (ΔG◦), enthalpy (ΔH◦), and entropy (ΔS◦) were calculated as follows:

(5) $${\Delta G}^{◦}=-RT\;\ln\;K_{c}$$

(6) $$K_{c}=\frac{q_{e}}{C_{e}}$$

(7) $$\ln\;K_{c}=\frac{{\Delta S}^{◦}}{R}-\;\frac{{\Delta H}^{◦}}{RT}$$

Kc is the distribution coefficient (Kc = Qe/Ce), ΔG◦ is the Gibbs free energy change, T is the temperature (K), and R is the gas constant (8.3145 J/mol K). In Table S4, the ΔG◦ values are negative at different temperatures, suggesting that the process is spontaneous. Increased temperature indicates a greater probability of Co (II) being adsorbed at equilibrium. ΔS◦ and ΔH◦ values were calculated from the van't Hoff plot (ln Kc vs. 1/T) in Fig. S4. A positive value of ΔH◦ corresponds to endothermic adsorption, whereas a positive value of ΔS◦ indicates that randomness is increasing during the adsorption process [66].

### 3.6 Regeneration experiment

The reusability of an adsorbent is an essential property of the adsorption process. To evaluate the reusability of the mesoporous Co/Ni MOF adsorbent, a four-step adsorption and desorption cycle of Co (II) was performed from 74.53 % to 62.98 % and 71.32 to 58.98 %. For desorption, 2 mL of 0.01 M HNO_3_ was added to the Co (II) solution containing the adsorbent. ICP-OES measured the remaining ions after stirring the adsorbent in an ultrasonic bath for 20 min. Satisfactory results were obtained, as illustrated in Fig. S1. The high mesoporous Co/Ni MOF adsorption capacity for Co (II) is evident from the adsorption-desorption percentage observed in the four-recovery cycle.

### 3.7 Adsorption mechanism

**Fig. 8:**
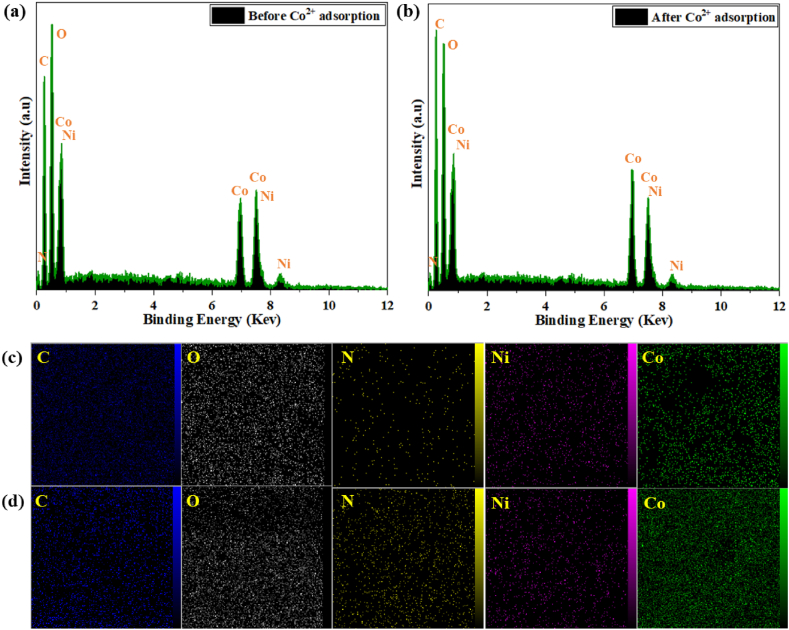
EDS spectra of mesoporous Co/Ni-MOF **(a)** Before adsorption Co (II), **(b)** After adsorption Co (II), **(c)** Elemental mapping of mesoporous Co/Ni-MOF before sorption process **(d)** Elemental mapping of mesoporous Co/Ni-MOF after Co (II) adsorption.

Various spectroscopic analyses were performed to investigate the mechanism of Co (II) adsorption on the MOF. The EDS spectra of mesoporous Co/Ni MOF were obtained before and after Co (II) adsorption. As shown in Fig. 8a and b, a new peak corresponding to the presence of Co (II) appeared, indicating the adsorption of the analyte on the surface of the adsorbent. Furthermore, elemental mapping of the MOF confirmed the presence of Co (II) within the structure after the adsorption process, as shown in Fig. 8c and d. The XRD patterns of the recycled MOF during the adsorption process showed no significant changes, indicating the structural stability of the MOF during the different stages of Co (II) adsorption. Similarly, the adsorbent's FT-IR spectra after the Co (II) solution uptake showed no significant changes, suggesting a physisorption mechanism for the adsorption of Co (II). However, slight changes in the position and shape of the peaks corresponding to C=O, C–H, and C–O bonds were observed in the FT-IR spectra (Fig. 3a), indicating the interaction between the linkers and the analyte, resulting in a decrease in the frequency and energy of the functional groups. Mesoporous Co/Ni MOFs adsorb Co (II) on their surfaces due to their organic ligand (terephthalic acid, pyrazine) interactions. There are two ways in which it can be performed: (I) surface coordination: Because Ni (NO_3_)_2_.6H_2_O and Co (NO_3_)_2_.6H_2_O have empty orbitals, Co (II) can coordinate with them on the surface. (II) mesoporous Co/Ni MOFs (pore size 38.7632 Å) are prepared, and Co (II) (ion radius of water coating = 6–7 Å) can create binding sites that affect the absorption of Co (II) [67,68].

## 4 Conclusions, future directions, and outlook

The study investigated a metal-organic framework (MOF) called mesoporous Co/Ni-MOF with terephthalic acid linkers, which was synthesized to adsorb cobalt ions from aqueous solutions. The adsorption tests conducted in this study showed that the adsorbent exhibited a favorable performance in adsorbing Co (II). Several parameters, including beginning Co (II) concentration, the effect of pH, duration time, and the amount of adsorbent, were evaluated to assess the adsorption capabilities of the mesoporous Co/Ni MOF. Remarkably, the adsorbent achieved Co (II) removal from the aqueous solution with an adsorption capacity of 372.66 mg g^−1^, which is relatively high. These results highlight the promising activity of the mesoporous Co/Ni MOF as an effective adsorbent for water treatment applications with satisfactory performance. These objectives will, however, require a significant amount of modification and development to achieve. (I) Surface modification: Add graphene oxide or carbon nanotube materials to the Co/Ni MOF surface to increase water pollutants' surface area and adsorption capacity. (II) The second aspect is to enhance the stability of the MOF by adding cross-linking agents or using linker molecules that are highly stable under harsh conditions. (III) A natural polymer, such as chitosan, alginate, or cellulose, can then be applied to the surface of the Co/Ni MOF. They can also improve the MOF's stability and biocompatibility while enhancing its adsorption capacity for water contaminants. (IV) The fourth method would be adding magnetic nanoparticles to the Co/Ni MOF structure, such as iron oxide (Fe_3_O_4_) or magnetite (Fe_2_O_3_). It is possible to easily separate and reuse the MOF with this modification, which enables it to be magnetically recoverable. (VI) Encapsulate Co/Ni MOFs within polymers containing magnetic nanoparticles to design core-shell structures. Water purification applications can benefit from this configuration because it can provide additional protection for the MOF while improving magnetic recovery and performance [[69], [70], [71]].

## Data availability

The data supporting this study's findings are available from the corresponding author, [Ali Maleki], upon reasonable request.

## CRediT authorship contribution statement

**Ehsan Moradi:** Writing – review & editing, Writing – original draft, Visualization, Validation, Supervision, Software, Resources, Methodology, Investigation, Funding acquisition, Formal analysis, Data curation, Conceptualization. **Mohammad Mehdi Salehi:** Writing – review & editing, Writing – original draft, Visualization, Validation, Supervision, Software, Resources, Methodology, Investigation, Funding acquisition, Formal analysis, Data curation, Conceptualization. **Ali Maleki:** Writing – review & editing, Writing – original draft, Visualization, Validation, Supervision, Software, Resources, Project administration, Methodology, Investigation, Funding acquisition, Formal analysis, Data curation, Conceptualization.

## Declaration of competing interest

All authors declare that they have no competing interests or any conflict of interests in this work.
