# Supplementary material for: Highly stable mesoporous Co/Ni mixed metal-organic framework [Co/Ni(μ3-tp)2(μ2-pyz)2] for Co (II) heavy metal ions (HMIs) remediation
Source: Heliyon. 2024 Jul 23;10(15):e35044. doi: 10.1016/j.heliyon.2024.e35044 (PMC11327570; doi:10.1016/j.heliyon.2024.e35044)
Supplement: Multimedia component 1 [file mmc1.docx]

**Highly Stable** **Mesoporous Co/Ni Mixed Metal-Organic Framework [Co/Ni(μ3-tp)_2_(μ2-pyz)_2_] for Co (II) Heavy Metal Ions (HMIs) Remediation**

Ehsan Moradi^1^, Mohammad Mehdi Salehi^1^, Ali Maleki^*^

*Catalysts and Organic Synthesis Research Laboratory, Department of Chemistry, Iran University of Science and Technology, Tehran 16846-13114, Iran.*

**Corresponding authors: E-mail: maleki@iust.ac.ir; Fax: +98-21-73021584; Tel: +98-21-73228313.*

*^1^ These authors have contributed equally in this work.*

*
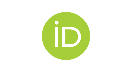
Author’s ORCIDs:*

*Ehsan Moradi:* [*https://orcid.org/0000-0002-8804-1856*](https://orcid.org/0000-0002-8804-1856)

*Mohammad Mehdi Salehi:* [*https://orcid.org/0000-0003-3648-1865*](https://orcid.org/0000-0003-3648-1865)

*Ali Maleki:* [*https://orcid.org/0000-0001-5490-3350*](https://orcid.org/0000-0001-5490-3350)

| **Content** | **Page** |
| --- | --- |
| **Table S1.** Materials and instruments used in this study | **S2** |
| **Table S2.** Comparison of the sorption capacities of various adsorbents for Co (II) ion removal | **S3** |
| **Table S3.** BET surface area, pore volume, and pore size of mesoporous Co/Ni-MOF | **S4** |
| **Table S4.** Isotherm and kinetic nonlinear and linear constants, correlation coefficients, and thermodynamics of adsorption of Co (II) on mesoporous Co/Ni-MOF | **S5** |
| **Table S5.** Linear and nonlinear isotherms and kinetic equations | **S9** |
| **Figure S1.** Adsorption-desorption cycles of Co/Ni-MOF | **S11** |
| **Figure S2.** Plots of linear and nonlinear isotherm models | **S12** |
| **Figure S3.** Plots of linear and nonlinear kinetic models | **S13** |
| **Figure S4.** Plots of Thermodynamics | **S14** |
| **References.** | **S15** |

Table S1. Materials and instruments used in this study

| Materials and Instruments | Brand and Purity |
| --- | --- |
| Ni (NO_3_)_2_.6H_2_O | Sigma Alderich, ≥98.5% |
| Terephthalic acid | Sigma Alderich (Cass No. 100-21-0) |
| Co (NO_3_)_2_^-.^6H_2_O | ACS reagent, ≥98% |
| Hydrochloric Acid (HCl) | Merck (37.0 %) |
| Sodium Chloride (NaCl) | ACS reagent, ≥99.0% |
| Philips X-pert diffractometer (PXRD) | Philips, Expert Pro, Netherlands |
| Fourier-Transform Infrared Spectroscopy (FT-IR) | AVATAR, Thermo Nicolet, spectrophotometer, USA |
| Thermogravimetric Analysis (TGA) | Bahr STA 504, Germany |
| Inductively Coupled Plasma Optical Emission Spectrometry (ICP-OES) | Shimadzu, ICP-7000 version II, Japan |
| Brunauer-Emmett-Teller (BET) | BET, Micrometics ASAP 2020, USA |
| Oven | Genlab Ltd, UK |
| Filter paper | Whatman (grade 602h, particle retention < 2µm) |
| Autoclave | Reyhan Teb, 2KW-220V, Iran |
| pH Meters | PHS-3C pH |
| BET | BELSORP-MINI II instrument, Japan |

Table S2. Comparison of the sorption capacities of various adsorbents for Co (II) ion removal

| MOFs | Adsorption capacity (mg.g^−1^) of Co (II) ions | Time to adsorption equilibrium (min) | Optimal pH | | Ref |
| --- | --- | --- | --- | --- | --- |
| ZIF-90-SO_2_HN_2_ | 122.85 | 120 | 6.72 | | [1] |
| UiO-66-COOCH_3_  UiO-66-CONH_2_  UiO-66-CN  UiO-66-SO_3_H | 334.4  339.7  274.6  293.7 | 2250 | 9 | | [2] |
| ZIF-8 | 178.15 | 720 | 7 | | [3] |
| MIL-100 (Fe) | 119 | 720 | 9 | | [4] |
| MCS/AC@UiO-66 | 44.5 | 15 | 8 | | [5] |
| ZIF-90-Met  ZIF-90-Lys | 136.83  164.40 | 140  140 | 8  8 | | [6] |
| Mesoporous silica | 6.62 | 25 | 7 | | [7] |
| GO-NH_2_ | 116.35 | 180 | 6 | | [8] |
| GC hydrogel | 202 | 180 | 7 | | [9] |
| DCPD | 441 | 1500 | 7 | | [10] |
| GG/SLS | 601 | 225 | 7 | | [11] |
| CpAD | 185.23 | 50 | 8 | | [12] |
| n-MgFe_2_O_4_ | 67.41 | 140 | 8 | | [13] |
| CTS  CCTS | 30.45  59.51 | - | 4.5  4.5 | | [14] |
| EDTA-chitosan | 61.0 | 400 | 7 | | [15] |
| Mesoporous Co/Ni MOF | 372.66 | 25 | 6 | This Work | |

Table S3. BET surface area, pore volume, and pore size of mesoporous Co/Ni-MOF

| Sample | Surface Area^a^ (m^2^.g^-1^) | Pore Volume^b^ (cm^3^.g^-1^) | Pore Size^b^ (nm) |
| --- | --- | --- | --- |
| Mesoporous Co/Ni-MOF | 2.3070 | 0.0022 | 38.7632 |

^a:^ The surface area parameter was acquired via BET analysis.

^b^: The pore volume and pore size parameters were acquired via BJH analysis.

**Table S4.** Isotherm and kinetic nonlinear and linear constants, correlation coefficients, and thermodynamics of adsorption of Co (II) on mesoporous Co/Ni-MOF

| Model | | Parameters | Co (II) |
| --- | --- | --- | --- |
| Isotherm | Linear Freundlich | K_F_ (mg.g^-1^) | 80.3185 |
|  |  | n | 2.613696 |
|  |  | R^2^ | 0.9509 |
|  | Linear Langmuir | Q_max, Cal_ (mg.g^-1^) | 370.3704 |
|  |  | K_L_ (L/mg) | 0.147541 |
|  |  | R^2^ | 0.896 |
|  | Nonlinear Freundlich | K_F_, mg.g^-1^ | 3.917491 |
|  |  | n | 1.3211 |
|  |  | R^2^ | 0.9999 |
|  | Nonlinear Langmuir | Q_max_ (mg.g^-1^) | 302.7408 |
|  |  | K_L_ (L/mg) | 0.2453 |
|  |  | R^2^ | 0.8644 |
|  | Linear Temkin | BT | 69.442 |
|  |  | KT | 2.319 |
|  |  | R^2^ | 0.8672 |
|  | Nonlinear Temkin | A | 2.3141 |
|  |  | B | 69.4233 |
|  |  | R^2^ | 1.00 |
|  | Linear Redlich Peterson (RP) | A | 78.319 |
|  |  | B | 0.6101 |
|  |  | R^2^ | 0.9806 |
|  | Nonlinear Redlich-Peterson (RP) | K | 18.730 |
|  |  | n | 2.021 |
|  |  | a | 0.0008 |
|  |  | R^2^ | 0.9746 |
| Kinetics | Linear Pseudo-first-order (PFO) | K_1_ (min^-1^) | 0.0713 |
|  |  | Qe, _experimental_ (mg.g^-1^) | 237.43 |
|  |  | Qe, _calculated_ (mg.g^-1^) | 92.64 |
|  |  | R^2^ | 0.9194 |
|  | Nonlinear Pseudo-first-order (PFO) | K_1_ (min^-1^) | 0.3898 |
|  |  | Q_e, experimental_ (mg.g^-1^) | 237.43 |
|  |  | Q_e, calculated_ (mg.g^-1^) | 209.634 |
|  |  | R^2^ | 0.999995 |
|  | Linear Pseudo-second-order (PSO) | k_2_ (min^-1^) | 0.0014 |
|  |  | Q_e, experimental_ (mg.g^-1^) | 237.43 |
|  |  | Q_e, calculated_ (mg.g^-1^) | 250 |
|  |  | R^2^ | 0.9879 |
|  | Nonlinear Pseudo-second-order (PSO) | k_2_ (min^-1^) | 0.0021 |
|  |  | Q_e, experimental_ (mg.g^-1^) | 237.43 |
|  |  | Q_e, calculated_ (mg.g^-1^) | 242.741 |
|  |  | R^2^ | 0.988409 |
|  | Linear Weber-Morris Intraparticle Diffusion | C | 7.1194 |
|  |  | Kdiff | 0.0535 |
|  |  | R^2^ | 0.8657 |
|  | Nonlinear Weber-Morris Intraparticle Diffusion | C | 41.8526 |
|  |  | Kdiff | 39.5597 |
|  |  | R^2^ | 0.8571 |
|  | Linear Elovich | α | 89733.55 |
|  |  | β | 0.035 |
|  |  | R^2^ | 0.8114 |
|  | Nonlinear Elovich | α | 0.653 |
|  |  | β | 0.2924 |
|  |  | R^2^ | 0.8784 |
| Thermodynamics |  | ΔGº= -5.61959 kJ.mol^-1^(T=288K)  ΔGº= -7.21846 kJ.mol^-1^ (T=318K)  ΔHº= -18.2035 kJ.mol^-1^  ΔSº= -79.94327 J.mol^-1^K^-1^ | |

**Table S5**. Linear and nonlinear isotherms and kinetic equations

| Model | | Linear and Nonlinear Equations |
| --- | --- | --- |
| Isotherm | Linear Freundlich | $lnq_{e}=lnK_{F}+\frac{1}{n}lnC_{e}$  $lnq_{e}vs lnC_{e}$ |
|  | Nonlinear Freundlich | $q_{e}=K_{F}{C_{e}}^{1/n}$  $q_{e}vs C_{e}$ |
|  | Linear Langmuir | $\frac{c_{e}}{q_{e}}=\frac{1}{K_{L} q_{max}}+\frac{1}{q_{max}}C_{e}$  $\frac{C_{e}}{q_{e}} vs C_{e}$ |
|  | Nonlinear Langmuir | $q_{e}=q_{m}K_{L}\frac{C_{e}}{{1+K}_{L} C_{e}}$  $q_{e}vs C_{e}$ |
|  | Linear Temkin | $q_{e}=\frac{RT}{b}lnA+\frac{RT}{b}lnC_{e}$  $q_{e}vs lnC_{e}$ |
|  | Nonlinear Temkin | $q_{e}=\frac{RT}{b}lnAC_{e}$  $q_{e}vs C_{e}$ |
|  | Linear Redlich-Peterson | $ln\left( \frac{{AC}_{e}}{q_{e}}-1 \right)=lnB+\beta lnC_{e}$  $ln\left( \frac{C_{e}}{q_{e}} \right) vs {lnC}_{e}$ |
|  | Nonlinear Redlich-Peterson | $q_{e}=\frac{AC_{e}}{1+B C_{e}^{\beta}}$  $q_{e}vs C_{e}$ |
| Kinetics | Linear Pseudo-first-order | $ln\left( q_{e}-q_{t} \right)=lnq_{e}-k_{1}t$  $ln\left( q_{e}-q_{t} \right) vs t$ |
|  | Nonlinear Pseudo-first-order | $q_{t}=q_{e}\left( 1-e^{-k1t} \right)$  $q_{t} vs t$ |
|  | Linear Pseudo-second-order | $\frac{t}{q_{t}}=\frac{1}{k_{2}q_{e}^{2}}+\frac{1}{q_{e}}t$  t/$q_{t} vs t$ |
|  | Nonlinear Pseudo-second-order | $q_{t}=\frac{k_{2}q_{e}^{2}t}{1+k_{2}q_{e}t}$  $q_{t} vs t$ |
|  | Linear Weber-Morris Intraparticle Diffusion | $q_{t}=k_{id}t^{\frac{1}{2}}+C$  ${q_{t} vs t}^{\frac{1}{2}}$ |
|  | Nonlinear Weber-Morris Intraparticle Diffusion | $q_{t}=k_{t}tn+C$  $q_{t} vs t$ |
|  | Linear Elovich | $q_{t}=\frac{1}{\beta}\ln\left( \alpha\beta\right)+\frac{1}{\beta}\ln t$  $q_{t} vs t$ |
|  | Nonlinear Elovich | $q_{t}=\frac{1}{\beta}ln \left( 1+\alpha\beta t \right)$  $q_{t} vs t$ |


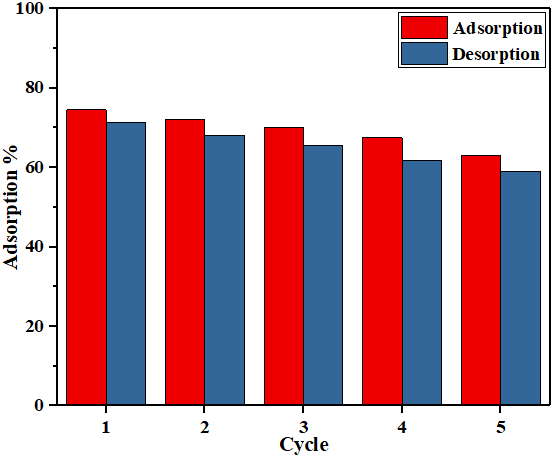


**Figure S1.** Adsorption-desorption cycles of mesopores Co/Ni-MOF (the percentage of desorption was calculated based on the amount of adsorption)


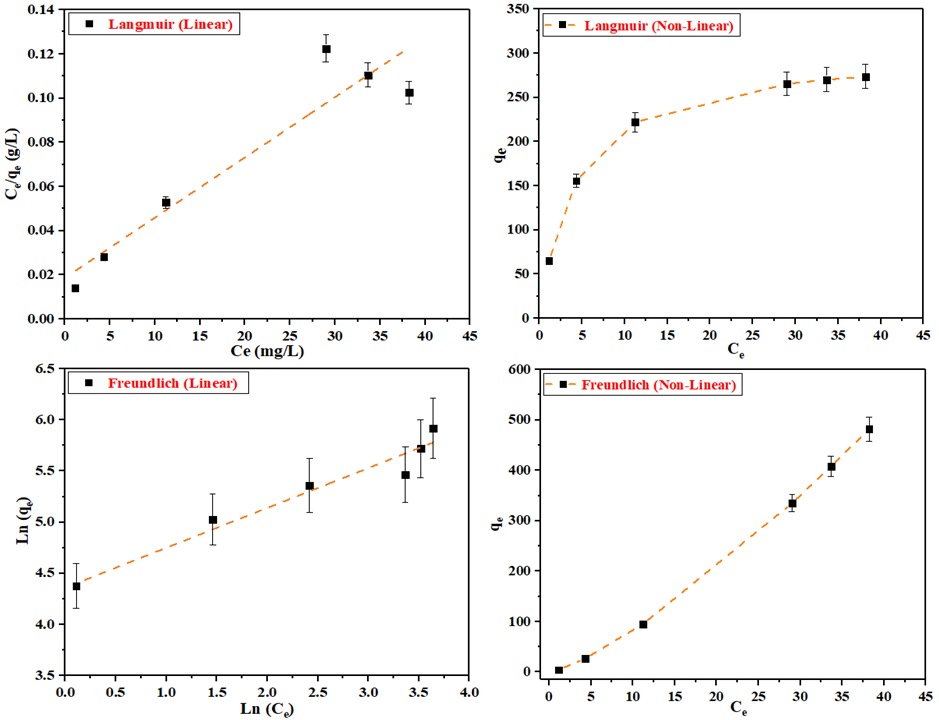


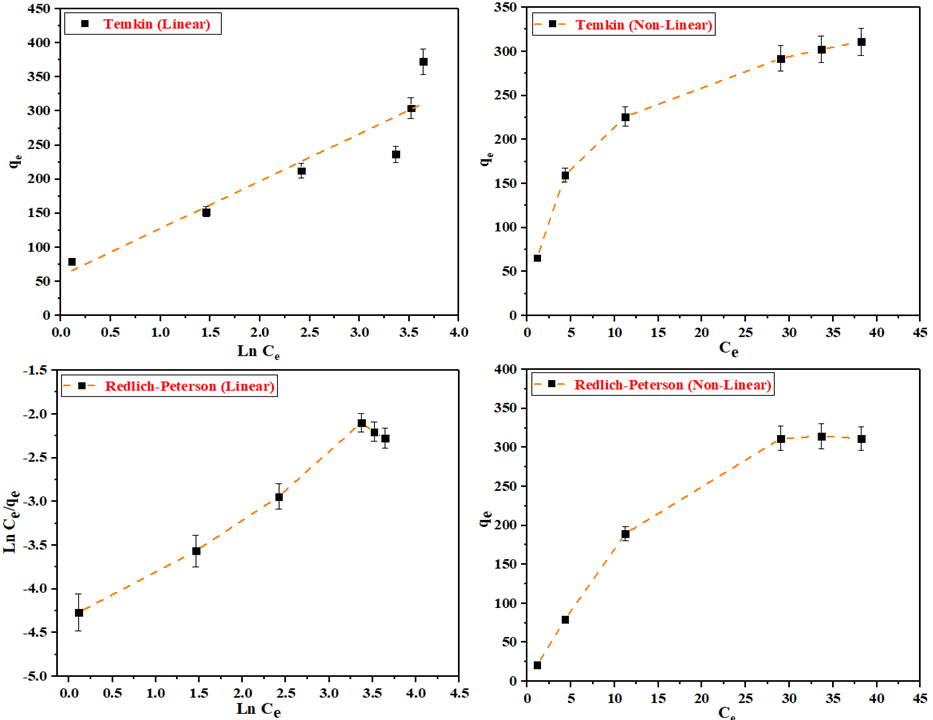


**Figure S2.** Plots of linear and nonlinear isotherm models.


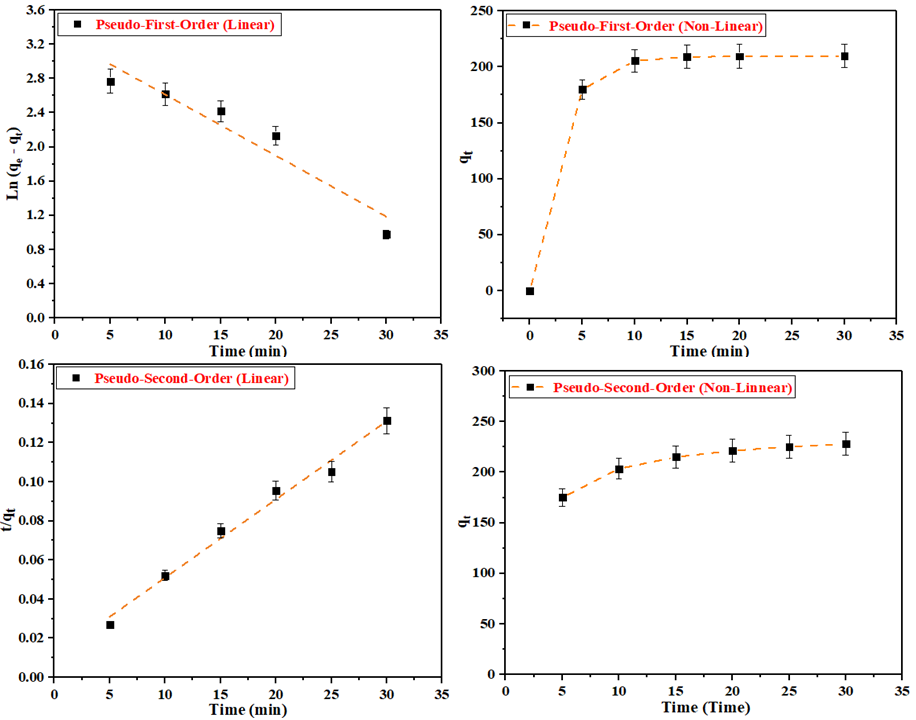


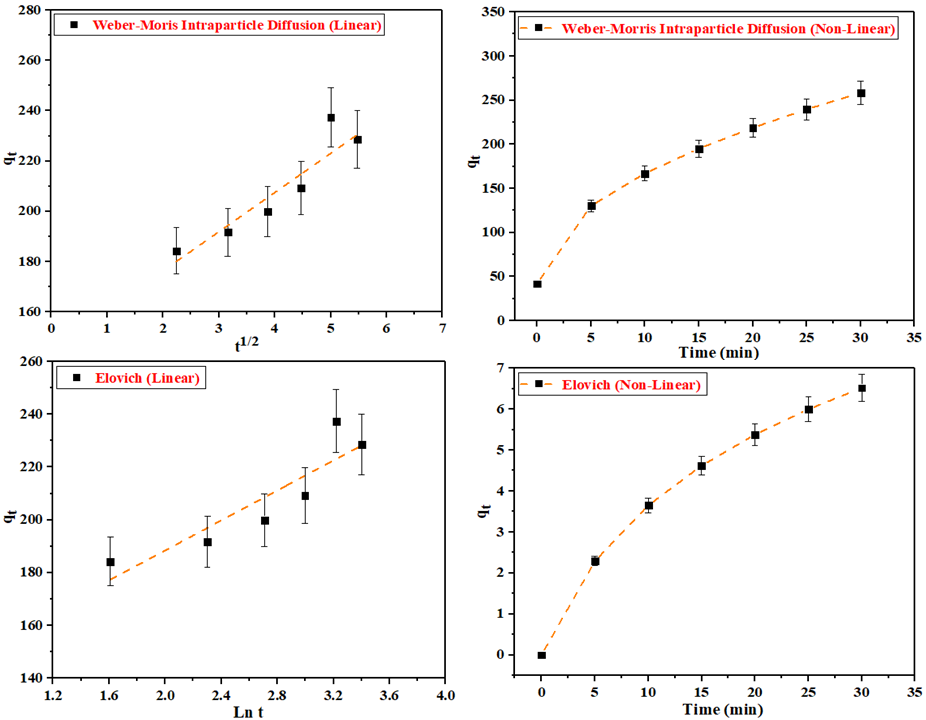


**Figure S3.** Plots of linear and nonlinear kinetic models.


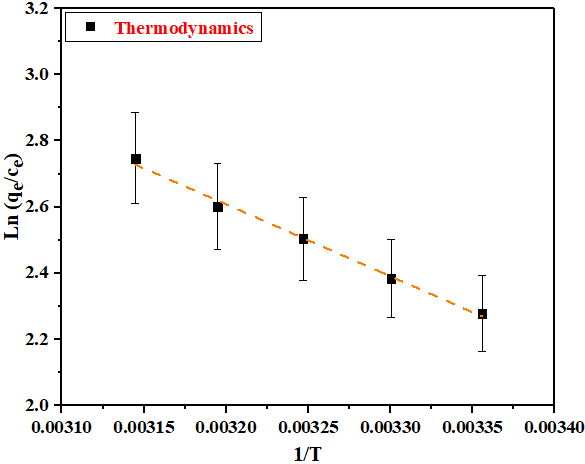


**Figure S4.** Plots of Thermodynamics.

***References:***

[1] Y. Zhou, X. Wang, J. Men, M. Jia, C. Liang, Adsorption performance of sulfonamide-modified metal–organic frameworks (MOFs) for Co (II) in aqueous solution, Journal of Radioanalytical and Nuclear Chemistry, 331 (2022) 3965-3977.

[2] G. Yuan, Y. Tian, M. Li, Y. Zeng, H. Tu, J. Liao, J. Yang, Y. Yang, N. Liu, Removal of Co (II) from aqueous solution with functionalized metal–organic frameworks (MOFs) composite, Journal of Radioanalytical and Nuclear Chemistry, 322 (2019) 827-838.

[3] Y. Zhou, X. Wang, J. Men, M. Jia, C. Liang, Study on the adsorption performance of zeolitic imidazolate framework-8 (ZIF-8) for Co2+ and Mn2+, Journal of Radioanalytical and Nuclear Chemistry, 331 (2022) 1367-1379.

[4] N. Abdollahi, G. Moussavi, S. Giannakis, A review of heavy metals’ removal from aqueous matrices by Metal-Organic Frameworks (MOFs): State-of-the art and recent advances, Journal of Environmental Chemical Engineering, 10 (2022) 107394.

[5] H. Motaghi, P. Arabkhani, M. Parvinnia, A. Asfaram, Simultaneous adsorption of cobalt ions, azo dye, and imidacloprid pesticide on the magnetic chitosan/activated carbon@ UiO-66 bio-nanocomposite: Optimization, mechanisms, regeneration, and application, Separation and Purification Technology, 284 (2022) 120258.

[6] X. Wang, Y. Zhou, J. Men, C. Liang, M. Jia, Removal of Co (II) from aqueous solutions with amino acid-modified hydrophilic metal-organic frameworks, Inorganica Chimica Acta, 547 (2023) 121337.

[7] M.H. Salmani, M.H. Ehrampoush, H. Eslami, B. Eftekhar, Synthesis, characterization and application of mesoporous silica in removal of cobalt ions from contaminated water, Groundwater for sustainable development, 11 (2020) 100425.

[8] F. Fang, L. Kong, J. Huang, S. Wu, K. Zhang, X. Wang, B. Sun, Z. Jin, J. Wang, X.-J. Huang, Removal of cobalt ions from aqueous solution by an amination graphene oxide nanocomposite, Journal of hazardous materials, 270 (2014) 1-10.

[9] Y. Liu, L. Hu, Y. Yao, Z. Su, S. Hu, Construction of composite chitosan-glucose hydrogel for adsorption of Co2+ ions, International journal of biological macromolecules, 139 (2019) 213-220.

[10] E.L. Vivas, K. Cho, Efficient adsorptive removal of Cobalt (II) ions from water by dicalcium phosphate dihydrate, Journal of environmental management, 283 (2021) 111990.

[11] X. Li, X. Wang, T. Han, C. Hao, S. Han, X. Fan, Synthesis of sodium lignosulfonate-guar gum composite hydrogel for the removal of Cu2+ and Co2+, International Journal of Biological Macromolecules, 175 (2021) 459-472.

[12] M.R. Awual, M.M. Hasan, A. Islam, A.M. Asiri, M.M. Rahman, Optimization of an innovative composited material for effective monitoring and removal of cobalt (II) from wastewater, Journal of Molecular Liquids, 298 (2020) 112035.

[13] V. Srivastava, Y. Sharma, M. Sillanpää, Application of nano-magnesso ferrite (n-MgFe2O4) for the removal of Co2+ ions from synthetic wastewater: kinetic, equilibrium and thermodynamic studies, Applied Surface Science, 338 (2015) 42-54.

[14] B. Liao, W.-y. Sun, N. Guo, S.-l. Ding, S.-j. Su, Comparison of Co2+ adsorption by chitosan and its triethylene-tetramine derivative: performance and mechanism, Carbohydrate polymers, 151 (2016) 20-28.

[15] S. Zhuang, Q. Zhang, J. Wang, Adsorption of Co2+ and Sr2+ from aqueous solution by chitosan grafted with EDTA, Journal of Molecular Liquids, 325 (2021) 115197.
